# COMP report: CPQR technical quality control guidelines for low‐dose‐rate permanent seed brachytherapy

**DOI:** 10.1002/acm2.12307

**Published:** 2018-03-14

**Authors:** Luc Beaulieu, Dee‐Ann Radford, J. Eduardo Villarreal‐Barajas

**Affiliations:** ^1^ Department of Physics Université Laval Cancer Research Centre Quebec QC Canada; ^2^ Department of Radiation Oncology CRCHU de Québec CHU de Québec – Université Laval Ville de Québec QC Canada; ^3^ Department of Oncology University of Calgary Calgary AB Canada; ^4^ Department of Medical Physics Tom Baker Cancer Centre Calgary AB Canada

**Keywords:** brachytherapy, low‐dose‐rate, quality control, seed implants

## Abstract

The Canadian Organization of Medical Physicists (COMP), in close partnership with the Canadian Partnership for Quality Radiotherapy (CPQR) has developed a series of Technical Quality Control (TQC) guidelines for radiation treatment equipment. These guidelines outline the performance objectives that equipment should meet in order to ensure an acceptable level of radiation treatment quality. The TQC guidelines have been rigorously reviewed and field tested in a variety of Canadian radiation treatment facilities. The development process enables rapid review and update to keep the guidelines current with changes in technology. This article contains detailed performance objectives and safety criteria for low‐dose‐rate (LDR) permanent seed brachytherapy.

## 1 INTRODUCTION

The Canadian Partnership for Quality Radiotherapy (CPQR) is an alliance among the national professional organizations involved in the delivery of radiation treatment in Canada: the Canadian Association of Radiation Oncology (CARO), the Canadian Organization of Medical Physicists (COMP), and the Canadian Association of Medical Radiation Technologists (CAMRT). Financial and strategic backing is provided by the federal government through the Canadian Partnership Against Cancer (CPAC), a national resource for advancing cancer prevention and treatment. The mandate of the CPQR is to support the universal availability of high quality and safe radiotherapy for all Canadians through system performance improvement and the development of consensus‐based guidelines and indicators to aid in radiation treatment program development and evaluation.

This document contains detailed performance objectives and safety criteria for *low‐dose‐rate Permanent Seed Brachytherapy*. Please refer to the overarching document *Technical Quality Control Guidelines for Canadian Radiation Treatment Centres* 1 for a programmatic overview of technical quality control, and a description of how the performance objectives and criteria listed in this document should be interpreted. This overall process is based on prior work by Dunscombe et al.^2^

The development of the individual TQC guidelines, this one included, is spearheaded by expert practitioners and involves broad stakeholder input from the medical physics and radiation oncology community.^3^ All information contained in this document is intended to be used at the discretion of each individual center to help guide quality and safety program improvement. There are no legal standards supporting this document; specific federal or provincial regulations and licence conditions take precedence over the content of this document.

## 2 SYSTEM DESCRIPTION

There are several other publications dealing with the performance, specifications, and quality control of low‐dose‐rate (LDR) permanent seed brachytherapy.^4^, ^5^, ^6^, ^7^, ^8^, ^9^, ^10^, ^11^ Most of these publications have extensive reference lists. Some have detailed descriptions indicating how to conduct the various quality control tests. The guidelines promoted in this document are based on the experience of the authors and expert practitioners and are broadly consistent with recommendations from other jurisdictions.^6^, ^7^, ^8^, ^9^, ^10^, ^11^

Brachytherapy is a procedure in which sealed radionuclide sources are placed in close proximity to, or inside, the tumor. For example, brachytherapy modalities for prostate cancer presently used in Canada include ultrasound guided transperineal interstitial permanent prostate brachytherapy (TIPPB) and high dose rate (HDR) brachytherapy. In prostate brachytherapy, four radionuclides are currently used: ^125^I, ^103^Pd, ^131^Cs, and, ^192^Ir. ^192^Ir is used for HDR brachytherapy. Quality control procedures are similar to those of other HDR procedures and can be found in the CPQR Technical Quality Control (TQC) guideline *Brachytherapy Remote Afterloaders*.^12^ ^131^Cs, ^125^I, and, ^103^Pd are used for permanent implants and are the radionuclides of interest here.

Transperineal interstitial permanent prostate brachytherapy was first proposed by Holm and colleagues.^13^ The procedure consists of using a transrectal ultrasound probe to first define the prostate contours in 1–5 mm‐thick transaxial images for dosimetric planning and then, some weeks later, delivering radioactive seeds (sources 0.8 mm in diameter × 4.5–5 mm in length) into the prostate gland. In both steps, the patient is placed in the lithotomy position. Over the years, other approaches have been introduced such as intra‐operative pre‐planning and interactive planning.^10^ In such cases, treatment planning and seeds loading in needles take place during the operative procedure. Needles containing the seeds are inserted through the perineum and into the prostate under the guidance of the transrectal ultrasound probe. The needles are prepared for the procedure in one of three ways: manual loading on site, purchased pre‐loaded needles, and seed loading devices. Some customization of the quality control guidelines presented here may be necessary to accommodate the particular method of needle loading in use.

Transperineal interstitial permanent prostate brachytherapy has become a very popular treatment alternative for low risk prostate cancer patients due to the pioneering work of the Seattle group.^14^ This treatment option is offered to patients having early localized prostate cancer (Stage < T2c, Gleason score < 7, and PSA < 10). Biochemical disease‐free survival rates have now been reported for this procedure for extended follow‐up periods.^14^, ^15^, ^16^, ^17^, ^18^, ^19^, ^20^, ^21^ Similar results are also available in a Canadian context.^22^, ^23^, ^24^, ^25^

For intermediate and high‐risk patients (PSA > 10 and/or Gleason score > 6 and/or stage > T2c), HDR brachytherapy is more commonly used, mainly as a boost strategy, producing excellent PSA control and negative biopsy results in patients with intermediate‐ and high‐risk prostate cancer.^26^, ^27^, ^28^ However, TIPPB alone is a treatment option for some low‐tier intermediate risk prostate cancer patients and can also be used as a boost modality.^23^, ^24^, ^25^

Recently, permanent seed implants have been proposed for breast cancer by Dr. Jean‐Philippe Pignol from Sunnybrook hospital in Toronto.^29^ The general guidelines described in this document and the literature review provided should enable the clinical physicists to adapt the standards set forth to that procedure.

A brachytherapy program, whether it involves permanent seed implants or HDR temporary implants, requires the competencies of multiple health professionals to be efficient and productive. From the physicist's point of view, there is a convergence of many technologies into a single procedure. American Association of Physicists in Medicine (AAPM) Task Groups 43U1,^7^ 64,^6^ 137,^10^ 138,^11^ 186 30 as well as the American Brachytherapy Society 5, 31 and Groupe Européen de Curiethérapie (GEC) and the European Society for Radiotherapy and Oncology (GEC‐ESTRO) guidelines,^8^ are reference documents for these procedures. The three areas of importance for all implants are: imaging, dosimetry, and radiation protection. Furthermore, general treatment planning systems (TPS) and Brachytherapy Task Group reports are also relevant as reference materials for the practicing clinical physicists. These include the AAPM Task Groups 40, 53, 56, 59, and 60.^32^, ^33^, ^34^, ^35^, ^36^

Furthermore, prostate brachytherapy is based first on the use of ultrasound as a real‐time guidance device. The AAPM has published a report from Task Group 128 dedicated to prostate brachytherapy ultrasounds quality assurance tasks.^9^

Conventional x ray films or fluoroscopy can also be used to visualize the seeds or the catheters after they have been implanted. Such verification can be made in the operating room or the brachytherapy suite. Finally, CT and MRI scans are used for TIPPB post‐plan quality assurance. For all prostate brachytherapy programs, a calibrated well chamber and hand‐held radiation monitor must be available at all times. Personal whole‐body dosimeters should be worn by all staff participating in the implant procedure. Other personal dosimeters, such as ring and wrist dosimeters, can also be used.

The dosimetric description of the sources should be made according to AAPM Task Group 43 recommendation.^7^, ^37^ The AAPM and the Imaging and Radiation Oncology Core (IROC) jointly maintain a registry of low‐energy brachytherapy seed designs that meet the AAPM dosimetric prerequisites. Peer reviewed articles giving dosimetric parameters of each of these seeds can be found in the registry (http://rpc.mdanderson.org/RPC/), along with a description of the AAPM prerequisites. The medical physicist should regularly carry out a thorough search of the scientific literature for any new assessment of a seed's dosimetric parameters and its potential impact on clinical dosimetry. While the literature does point out the limitations to TG43 with regards to procedures described in this document (mainly non‐water equivalent tissues as well as inter‐seed attenuation), no commercial solution is available to the clinical users and therefore are not covered herein. The interested readers should refer to the Task Group 186 report for more details.^30^

Any new or upgraded TPS and/or new seed model should be validated against known test cases and also by hand calculation. Potentially helpful in this regard are the test cases used by the Radiological Physics Center (RPC) at the MD Anderson Cancer Center for credentialing participants in clinical trials research having an LDR brachytherapy component. See the “Credentialing” section of the IROC website (http://rpc.mdanderson.org/RPC/). Before using a seed model clinically for the first time, a well chamber should be sent to an accredited dosimetry calibration laboratory (ADCL) for calibration. Alternatively, a single seed can be sent to an ADCL for measurement of its air‐kerma strength, and this value used to obtain a calibration factor for the well chamber. Compliance with applicable radiation safety codes must be ensured for each radionuclide, source type, and activity range to be used.

## 3 RELATED TECHNICAL QUALITY CONTROL GUIDELINES

In order to comprehensively assess low‐dose‐rate brachytherapy system performance, additional guideline tests, as outlined in related CPQR TQC guidelines must also be completed and documented, as applicable. Related TQC guidelines, available at cpqr.ca, include:

- Safety Systems.
- Major Dosimetry Equipment.

## 4 SEED‐IMPLANT‐SPECIFIC TEST TABLES

For LDR permanent seed brachytherapy, tests are required for mechanical, radiological, and safety systems. The minimum recommendations for LDR permanent seed brachytherapy quality control are listed in Tables 1 and 2. These guidelines consist of a series of tests to be performed, along with their minimum frequency. The tests are derived from the published literature and, in particular, are the standards laid out in the AAPM documents described previously.

**Table 1:** Daily quality control tests

Designator	Test	Performance	
		Tolerance	Action
Daily			
DPB1	Radiation survey meter	Functional	
DPB2	Source strength verification (well chamber)	3%	5%
DPB3	Ultrasound system/probe	Functional	
DPB4	Source inventory	Complete	
DPB5	Records	Complete	
DPB6	Room survey (drape, needle, template, etc.) *or* planning and seed loading devices	Complete	
DPB7	Console displays (treatment status indicator, date, time) *– if applicable, see text*	Functional	
DPB8	Printer operation, paper supply *– if applicable, see text*	Functional	
DPB9	System self‐test *– if applicable, see text*	Functional	
DPB10	Delivery interrupt *– if applicable, see text*	Functional	
DPB11	Power failure recovery *– if applicable, see text*	Functional	
DPB12	Data transfer from planning computer *– if applicable, see text*	Functional	
DPB13	Seed loading devices and disposable elements *– if applicable, see text*	Functional	
DPB14	Communication between all systems *– if applicable, see text*	Functional	
DPB15	Emergency seed loading kit *– if applicable, see text*	Functional/Sterilized	
DPB16	Online source strength verification *– if applicable, see text*	8%	15%
DPB17	Needle loading sequence as per treatment plan	Complete	

**Table 2:** Annual and bi‐annual quality control tests

Designator	Test	Performance	
		Tolerance	Action
Annually			
APB1	Ultrasound positional accuracy	1 mm	2 mm
APB2	Ultrasound volumetric accuracy	3%	5%
APB3	Stepper positional accuracy	1 mm	2 mm
APB4	Template positional accuracy	1 mm	3 mm
APB5	Source parameters and TPS dose calculation verification	2%	3%
APB6	Emergency seed handling procedures review	Complete	
APB7	Independent quality control review	Complete	
APB8	**End‐to‐end** system validation *or* planning and seed loading devices	Functional	
APB9	Online source strength measurements device calibration/verification *‐ if applicable*	3%	5%
APB10	Source positional accuracy (loading devices) *‐ if applicable*	2 mm	3 mm
APB11	Survey meter calibration	Complete	
Bi‐annually			
BPB1	Well‐chamber calibration	1%	2%

Any maintenance on the ultrasound, treatment planning computer, seed loading devices, and so on should be followed by thorough quality assurance testing involving the daily and/or annual quality assurance appropriate to the situation.

For seed implants, some of the daily tests should be performed either before each procedure (before each implant) or once at the start of the day, depending of the nature of the test.

Radiation safety related tests have not been included in Tables 1 and 2 but must be part of a comprehensive quality assurance program (see CPQR's companion guidance document *Quality Assurance Guidelines for Canadian Radiation Treatment Programs* 38). Specific license requirements and applicable safety codes must be followed. For example, Canadian Nuclear Safety Commission (CNSC) annual documentation and report for manual and afterloading brachytherapy must be performed. Furthermore, the quality assurance of imaging devices used as part of seed implant procedures (C‐arm, cone beam CT [CBCT], CT, US, and MRI scanners) must be performed according to the devices’ protocol and are not covered in this document.

### Notes on daily tests.

**Table:** 

DPB1	Verify that the handheld radiation survey meter (e.g., *Geiger counter*) is functional.
DPB2	The AAPM Low Energy Brachytherapy Source Calibration Working Group has outlined specific criteria.^39^ In general 10% of the seeds or 10 seeds, whichever number is larger, should be tested. For a sterile assembly, such as a sterile seed cartridge or pre‐loaded needle, the recommendation is the lowest of 5% of the seeds or 5 seeds. Complete descriptions of the scenarios between these two extremes are given in “table 1” of Butler et al., 2008.^39^ Remember that manufacturers usually ship seed strength within a range that can be as large as ±4% of the average strength.^11^ In addition to the above, a secondary device can be further used as part of a seed loader (e.g., Isoloader from Mentor or SeedSelectron from Nucletron) for which more than 10% and up to 100% of the seeds can be measured. Validation studies of the Isoloader ^40^ and SeedSelectron ^41^ have been published.
DPB3	In addition, visually inspect images for any artifacts, such as black lines or bands. Ensure they are not due to poor contact between the probe and tissue. If present, such bands may indicate non‐functioning ultrasound detector elements within the probe. Persistence of these artifacts may warrant image quality tests using a dedicated ultrasound phantom to characterize the location of the signal dropout and identify non‐functioning elements within the probe, which may have to be sent for repairs.
DPB4	Could be performed in conjunction with DPB2 above if done on the same day as the procedure. Otherwise, inventory should be validated before moving the sources to the procedure room.
DPB5	Documentation relating to the daily quality control checks, preventive maintenance, service calls, and subsequent checks must be complete and legible. The operator(s) must be identified.
DPB6	The workspace (including the floor), needles, template, probes, etc., must be surveyed using a calibrated survey meter (see DPB1). Reading should be consistent with no radioactive materials outside the seeds implanted in the patient. This task must be performed after each implant.
DPB7–15	The configuration of these tests will depend on the equipment selected and the clinical workflow (pre‐planning/live planning with or without a seed loading device). Safety is the concern and tests should be designed accordingly. As a minimum, manufacturer's recommendations and applicable regulations must be followed.
DPB16	See DPB2 above regarding detector such as the SeedSelectron.
DPB17	It is crucial that the needle loading sequence of each needle composing a given plan be validated and correspond to the treatment plan. For pre‐loaded needles, auto‐radiograph or x‐ray imaging will confirm the seed‐spacer sequence (or seed sequence for stranded seeds). For intra‐operative loading, a second person could visually confirm the loading as it is being done and most brachytherapy needle have graduation that can confirm the overall sequence length. In any case, this length should be confirmed (pre‐loaded or intra‐operative loading) before every needle insertion. Some devices, such as the SeedSelectron, have an array of radiation detectors that is used to confirm the seed (radiation present) and spacer (no radiation) sequence before loading. In such a case, the device itself should be regularly tested (see APB9 below).

### Notes on annual and bi‐annual tests.

**Table:** 

APB1–4	AAPM Task Group 128 constitutes the reference document with regard to ultrasound system performance and related quality assurance tasks; a detail description of each test is given.^9^ Transverse and longitudinal positional accuracy, as well as volume accuracy, can be measured using specially designed phantoms, (e.g., Computerized Imaging Reference Systems [CIRS] brachytherapy phantom model 45). Information about ultrasound verification procedures (e.g., use of ethylene glycol–water mixture and water temperature) for prostate can be found in Goldstein et al., 2002 ^42^ A simple prostate implant template verification setup is also described in Mutic et al., 2000 ^43^ In addition, various manufacturers also have their own recommendations. Please note that the speed of sound of tissue is 1540 m/s and phantom should mimic this property. Room temperature water‐like speed of sound is not acceptable (1482 m/s) for these tests.
APB5	Peer reviewed articles giving dosimetric parameters of each approved seed model can be found in the registry (http://rpc.mdanderson.org/rpc). The source data are usually based on Monte Carlo calculations *and* on experimental measurements, the combination being referred to as a consensus dataset.^7,37^ Validation of the parameters in the TPS can be performed in two ways: (1) a simple 1D hand calculation for a single source compared to the TPS or (2) a simple geometry involving a few seeds which can be reproduced in the TPS and in independent software (Excel, Matlab, or another commercial TPS). Tolerance and action levels refer to agreement between the TPS and an independent calculation. If another commercial TPS is used, validation of a reference structure volume can also be performed at the same time (volume handling can be a source of discrepancy between TPSs with regard to dose–volume histograms [DVHs]). Volumes between the two TPSs should agree within 5%.
APB6	The configuration of these tests will depend on the design of the facility and equipment used. Review the emergency procedures for seed/needle loading if a seed loading device is normally used and fails. Emergency procedures (e.g., if a seed should drop on the floor, is stuck in a needle, or is found in the urine bag) should be reviewed.
APB7	To ensure redundancy and adequate monitoring, a second qualified medical physicist must independently verify the implementation, analysis, and interpretation of the quality control tests at least annually.
APB8	It is recommended that a complete system validation be conducted once a year. In the present document this would include all the necessary validation for full system recovery from power outage (planning system recovery, seed delivery system, etc.,), delivery interruption, and other potentially deleterious events, as indicated in DBP6 to DPB13. These tests should be performed away from the daily clinical pressure and busy operating room environment.
APB9, 10	These measurements have been discussed in various publications.^40,41^
APB11	Survey meter should be calibrated once every 12 months as per CNSC requirements (Nuclear Substances and Radiation Devices Regulations [SOR/2000‐207]^44^).
BPB1	The well chamber should be sent to an accredited dosimetry calibration laboratory once every 2 years. A calibrated source, of each seed model used, could also be acquired from the manufacturer each year for verification purposes.

## CONFLICT OF INTEREST

The authors declare no conflict of interest.
